# The 2024 resurgence of *Bordetella pertussis* in Brazil and a decade-long epidemiological overview

**DOI:** 10.3389/fpubh.2025.1549735

**Published:** 2025-05-27

**Authors:** Nathália Mariana Santos Sansone, Matheus Negri Boschiero, Fernando Augusto Lima Marson

**Affiliations:** 1 LunGuardian Research Group—Epidemiology of Respiratory and Infectious Diseases, São Francisco University, Bragança Paulista, Brazil; 2 Laboratory of Molecular Biology and Genetics, São Francisco University, Bragança Paulista, Brazil; 3 Laboratory of Clinical and Molecular Microbiology, São Francisco University, Bragança Paulista, Brazil; 4 Medical Resident of Infectious Diseases at the Federal University of São Paulo, São Paulo, Brazil

**Keywords:** *Bordetella pertussis*, epidemiology, incidence, pertussis, public health, vaccine

## Abstract

Pertussis, or whooping cough, is a highly contagious respiratory illness caused by *Bordetella pertussis*. It primarily affects humans by adhering to the cilia of the upper respiratory tract, releasing toxins that impair ciliary function, and inducing inflammation. It is transmitted through respiratory droplets, and the disease is particularly severe in infants under 1 year of age, often resulting in high morbidity and frequent admission to intensive care units. Globally, the incidence of pertussis has been increasing, with notable increases observed in countries such as the United States, Brazil, and Mexico. In Brazil, the number of reported cases increased sharply in 2024 to 7,438—with a 34.4-fold increase compared to 2023 (*N* = 216)—with 30 associated deaths, the first since 2021. While most cases still affect children under 14 years of age, infections among individuals over 15 years of age have also increased, suggesting a demographic shift. The number of hospitalizations increased from 236 in 2023 to 739 in 2024 (a 3.1-fold increase). Geographically, the South region recorded the highest number of cases (*N* = 3,579), followed by the Southeast (*N* = 3,134). Incidence rates mirrored this pattern, with the South reporting 11.5 cases per 100,000 inhabitants and the Southeast, 3.54 per 100,000 inhabitants. Despite a slight improvement in vaccination coverage, it remains below the World Health Organization’s recommended threshold of 90%. Vaccination—particularly with the pentavalent and diphtheria, tetanus, and acellular pertussis (DTaP) vaccines—remains a critical tool for outbreak prevention; however, waning immunity underscores the need for booster doses across age groups. The coronavirus disease 2019 (COVID-19) pandemic likely reduced transmission temporarily due to mitigation measures and also led to missed routine vaccinations. To address this resurgence, Brazil must prioritize increasing vaccination coverage, especially among children, strengthening epidemiological surveillance, and improving healthcare provider training in immunization practices.

## Introduction

Whooping cough, a highly contagious respiratory illness caused by *Bordetella pertussis*, can be readily transmitted through airborne routes. During episodes of sneezing or coughing, infected individuals release small respiratory droplets containing *Bordetella pertussis*, which can be inhaled by others, leading to new infections (1). Pertussis is highly severe in children under 1 year of age, with substantial morbidity and elevated rates of intensive care unit admission within this age group (2). Given the current epidemiological context, this study aims to describe the ongoing resurgence of *B. pertussis* in Brazil in 2024 and to provide a decade-long epidemiological overview.

### *Bordetella pertussis* scenario in Brazil

**Figure 1:**
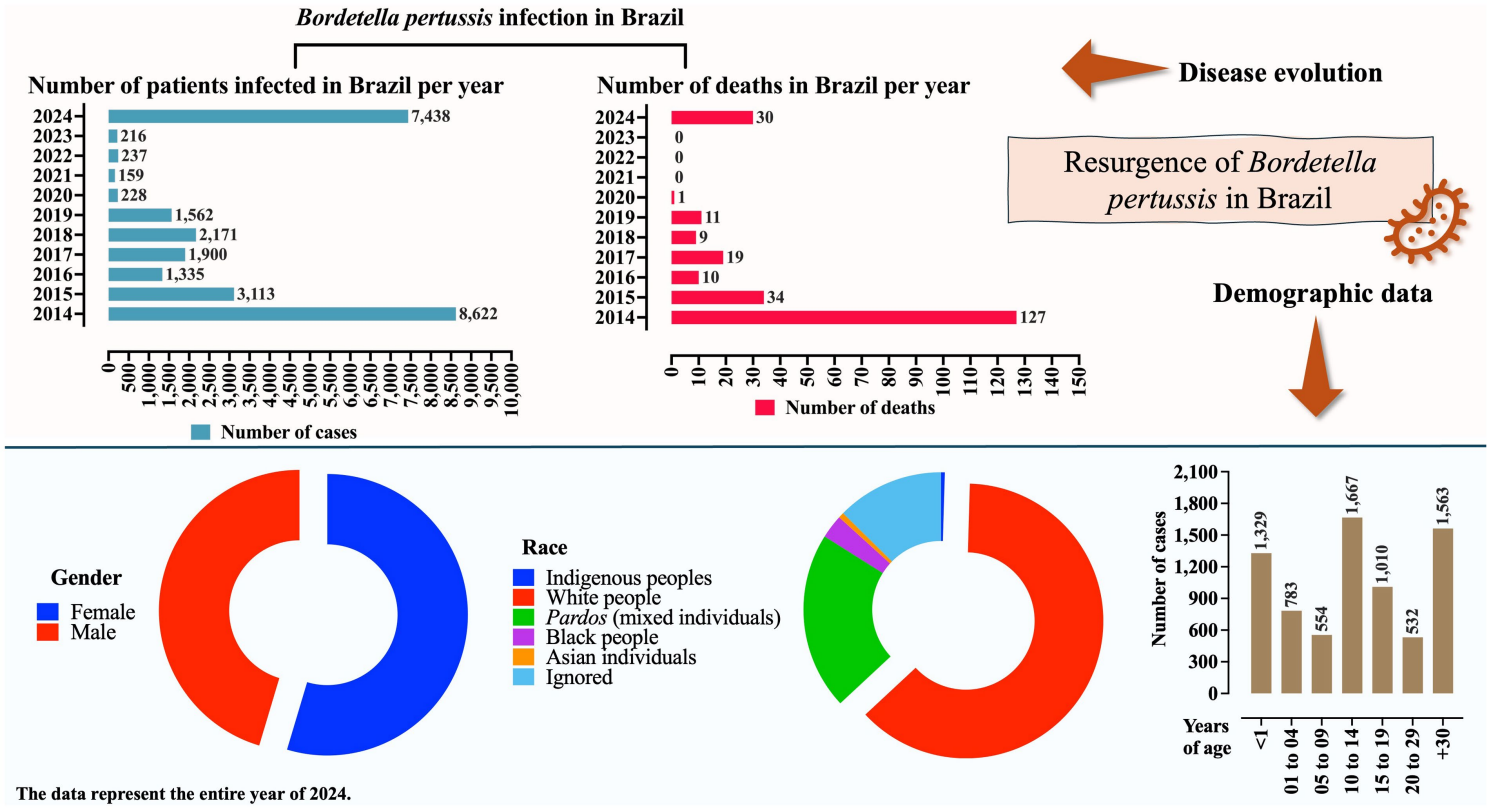
Profile of *Bordetella pertussis* infection in Brazil based on the number of cases, deaths, sex, race, and age groups. Data on sex, race, and age refer to the year 2024. Information is presented as the number of cases (*N*) or percentage. Epidemiological data on pertussis in Brazil were obtained from the Brazilian Ministry of Health (https://www.gov.br/saude/pt-br). Data were collected on 24 April 2025. Some changes may have occurred, particularly regarding the 2024 figures, due to the inclusion of newly confirmed cases.

Pertussis appears to be on the rise in several countries worldwide, with a notable increase reported in the United States and Latin American nations, including Brazil and Mexico (3). In 2024, data indicate a 300% increase in pertussis cases in the United States, whereas the incidence has increased by 242% in Mexico. Among Latin American countries, Brazil appears to be experiencing one of the most severe outbreaks, according to the Brazilian Ministry of Health. In 2024, a total of 7,438 pertussis cases were reported, with an incidence of 3.5 cases per 100,000 inhabitants, representing an increase of approximately 34.4 times compared to the total number of cases in 2023 (*N* = 216). Additionally, Brazil reported 30 pertussis-related deaths since 2024—the first of such fatalities since 2021 (4) (Figure 1). Figure 1 also depicts the distribution of patients among pertussis cases in 2024, according to sex, race, and age. In summary, most patients in that year were female, of White ethnicity, and over 14 years of age. The sex distribution was similar to the findings of previous years; however, the age distribution was notably different. In contrast to the participants in 2024, the majority of cases in previous years occurred in patients under 1 year of age (Supplementary Table 1).

**Figure 2:**
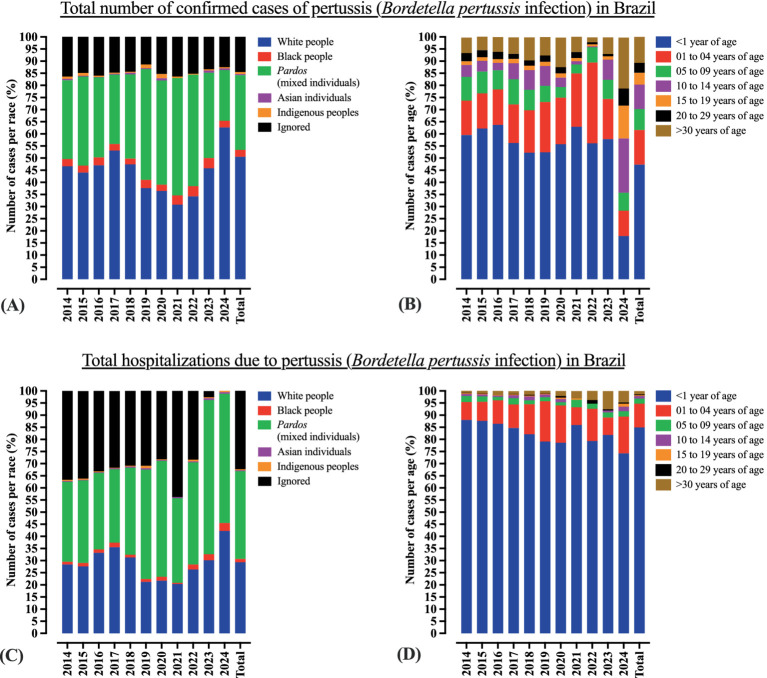
Profile of *B. pertussis* infection in Brazil by race and age. **(A)** Profile of *B. pertussis* infection in Brazil by race. **(B)** Profile of *B. pertussis* infection in Brazil by age. **(C)** Profile of *B. pertussis* infection among hospitalized patients in Brazil by race. **(D)** Profile of *B. pertussis* infection among hospitalized patients in Brazil by age. Data are presented as percentages. Complete data are provided in Supplementary Tables 1, 2. Epidemiological data on pertussis in Brazil were obtained from the Brazilian Ministry of Health (https://www.gov.br/saude/pt-br). Data were collected on April 24, 2025. Some changes may have occurred, particularly regarding the 2024 figures, due to the inclusion of newly confirmed cases.

Interestingly, the age distribution of pertussis cases shifted between 2023 and 2024. As expected, in both years, the majority of cases occurred in children aged 14 years or younger (196 cases [90.7%] in 2023 vs. 4,333 cases [58.3%] in 2024). However, a substantial increase was observed in 2024 among individuals aged ≥15 years (20 cases [9.2%] in 2023 vs. 3,105 cases [41.7%] in 2024) (Supplementary Table 1; Figure 2B). In terms of ethnicity, individuals identifying as White were more affected in 2024 (4,657 cases [62.6%]) compared to individuals affected in 2023 (99 cases [45.8%]), whereas the opposite trend was observed among individuals who identified as *Pardos,* who accounted for more cases in 2023 (76 cases [35.2%]) than in 2024 (1,553 cases [20.9%]) (Supplementary Table 1; Figure 2A). Supplementary Table 2 and Figure 2 present the trends in the number of cases by race and age group, respectively, from 2014 to 2024. It is important to note that the actual number of pertussis cases may be significantly underestimated. For example, in the United States, studies have suggested that the true incidence may be five to six times higher than the reported value. Similarly, in several Latin American countries, particularly among individuals over 50 years of age, the true incidence may be up to 100 times greater than that of the reported figures (5–7) (Figure 1).

The number of hospitalizations has increased from 236 in 2023 to 739 in 2024 (a 3.1-fold increase) (Supplementary Table 2). Although the majority of hospitalized cases in both years were infants under 1 year of age (193 cases [81.7%] in 2023 vs. 549 cases [74.2%] in 2024), an increase was observed among children aged 1–4 years in 2024 (17 cases [7.2%] vs. 112 cases [15.1%]). Additionally, there was an increase in the proportion of hospitalized White patients (71 cases [30.0%] in 2023 vs. 312 cases [42.2%] in 2024) and male patients (105 cases [44.5%] vs. 363 cases [49.1%], respectively). Although the overall epidemiological profile remained relatively consistent between 2023 and 2024, these subtle shifts should be taken into account when analyzing, predicting, and managing the ongoing resurgence. Supplementary Table 2 presents the age distribution (Figure 2D), sex, race (Figure 2C), and geographical location of the individuals hospitalized for *B. pertussis* infection in Brazil.

**Figure 3:**
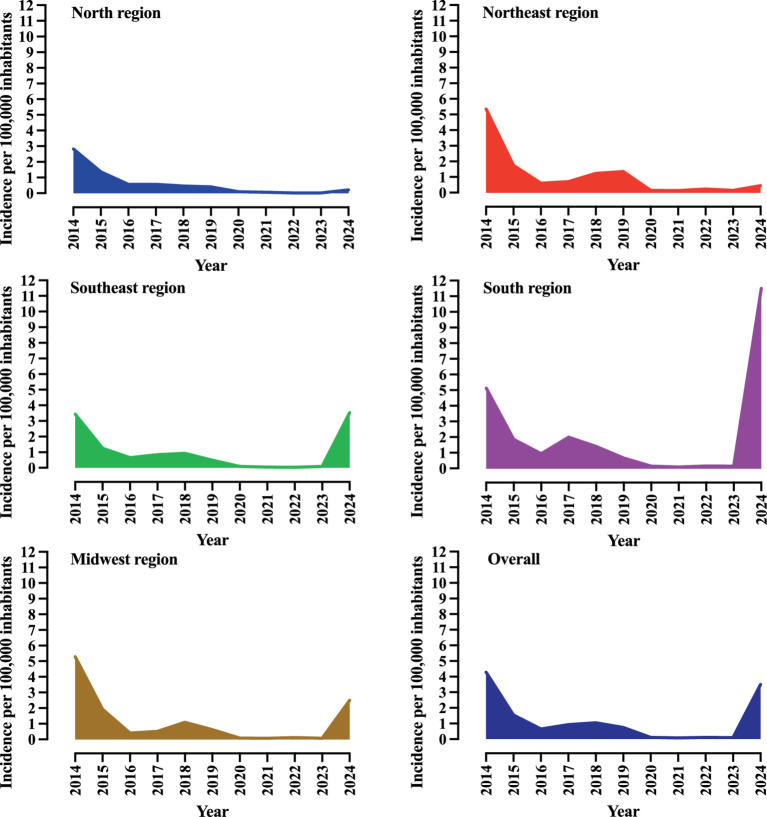
Incidence of *Bordetella pertussis* infection in Brazil by geographic macro-region over a 10-year period. Incidence is presented as the number of cases per 100,000 inhabitants. The complete data are provided in Supplementary Table 3. Epidemiological data on pertussis in Brazil were obtained from the Brazilian Ministry of Health (https://www.gov.br/saude/pt-br). Data were collected on 24 April 2025. Some changes may have occurred, particularly regarding the 2024 figures, due to the inclusion of newly confirmed cases.

Given Brazil’s continental dimensions, the distribution of pertussis cases across regions is heterogeneous. In 2024, the South recorded the highest number of cases (*N* = 3,579), followed by the Southeast (*N* = 3,134), Brazil’s most populous region. This finding contrasts with the pattern observed in 2023, when the Southeast region led in all cases (*N* = 75), followed by the Northeast (*N* = 83). When considering incidence, the distribution in 2024 remained consistent, with the South reporting 11.5 cases per 100,000 inhabitants and the Southeast reporting 3.54 per 100,000 inhabitants. All data are available in Supplementary Tables 3, 4 and Figure 3.

**Figure 4:**
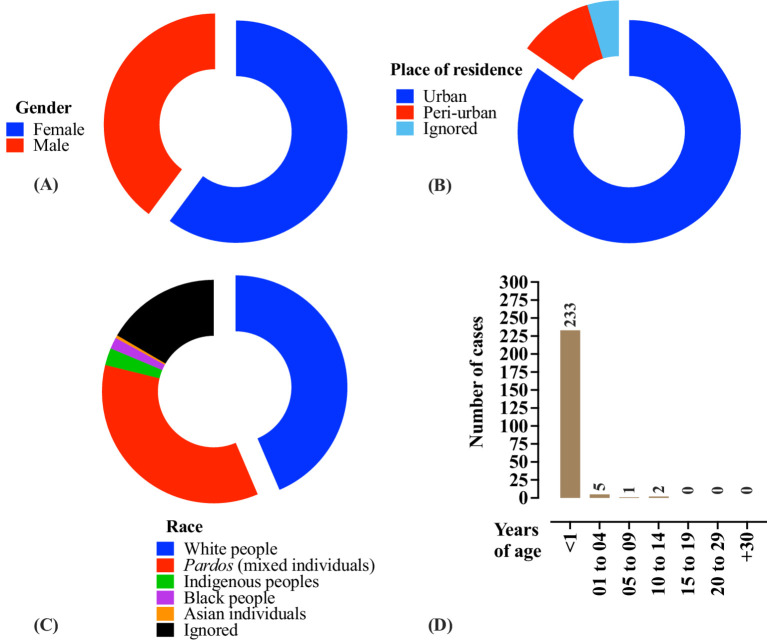
Profile of *B. pertussis*-related deaths in Brazil according to sex **(A)**, race **(B)**, place of residence **(C)**, and age **(D)**. The data correspond to the period between 2014 and 2024 and are presented as the number of cases (*N*) or percentages. Epidemiological data on pertussis in Brazil were obtained from the Brazilian Ministry of Health (https://www.gov.br/saude/pt-br). Data were collected on April 24, 2025. Some changes may have occurred, particularly regarding the 2024 figures, due to the inclusion of newly confirmed cases.

In Brazil, 241 deaths were attributed to pertussis cases between 2014 and 2024 (Figure 4). The majority of deaths occurred among female patients (*N* = 145 [60.0%]), patients residing in urban areas (*N* = 204 [84.6%]), and those who identified as White (*N* = 105 [43.5%]). Most fatalities (*N* = 233) occurred in infants under 1 year of age. Only eight deaths were reported in the other age groups (Figure 4). Epidemiological data on pertussis in Brazil were obtained from the Brazilian Ministry of Health.^1^

## Discussion

### *Bordetella pertussis* vaccination in Brazil: is it a successful public health initiative?

Pertussis prevention is primarily achieved through immunization. In Brazil, the pentavalent vaccine, which provides protection against diphtheria, tetanus, pertussis, hepatitis B (recombinant), and *Haemophilus influenzae* type *B* (conjugate), is offered free of charge by the Brazilian Unified Health System (*Sistema Único de Saúde*, SUS). The vaccination schedule included the first dose at 2 months of age, followed by additional doses at 4 and 6 months. The diphtheria, tetanus, and acellular pertussis (DTaP) vaccine was administered as a first booster at 15 months and a second booster at 4 years of age. Additionally, the SUS provides DTaP vaccination to all pregnant women regardless of their previous immunization status. Vaccination has proven effective in preventing outbreaks; however, periodic booster doses are necessary because vaccine-induced immunity wanes over time (8). Furthermore, since a natural infection does not confer lasting immunity, adults are recommended to receive DTaP booster doses every 5–10 years (9).

**Figure 5:**
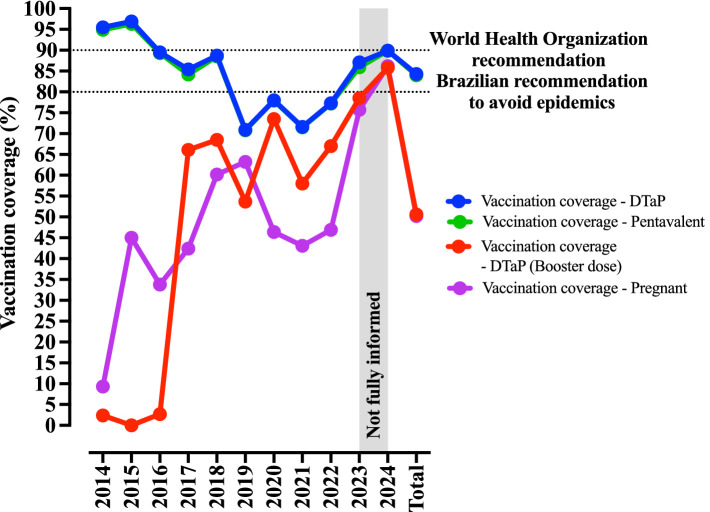
Vaccination profile against *B. pertussis* infection in Brazil. Data are presented as percentages of vaccination coverage. The pentavalent vaccine protects against diphtheria, tetanus, pertussis, hepatitis B (recombinant), and *Haemophilus influenzae* type *B* (conjugate). The DTaP vaccine protects against diphtheria, tetanus, and pertussis. Vaccination coverage for pregnant women was not available for 2024. Epidemiological data on pertussis in Brazil were obtained from the Brazilian Ministry of Health (https://www.gov.br/saude/pt-br).

The Brazilian Ministry of Health recommends maintaining vaccination coverage above 80% to prevent epidemics. In 2022, the coverage rates for the DTaP vaccine, first DTaP booster, pentavalent vaccine, and maternal immunization were 77.3, 67.0, 77.2, and 46.9%, respectively. By 2024, these rates had increased to 87.1, 78.5, 85.9, and 75.7%, respectively (Figure 5) (10). Unfortunately, the Brazilian Ministry of Health does not provide publicly available data on the vaccination status of confirmed pertussis cases, which limits the depth of analysis. Nevertheless, it is reasonable to assume that most deaths and complications occur among unvaccinated individuals, as the DTaP vaccination has been associated with a reduction in infant mortality and hospitalizations due to whooping cough (11).

Vaccine hesitancy is primarily driven by cultural factors, including the spread of misinformation regarding adverse effects and the limited understanding of the true severity of the disease. Although most countries have established national immunization programs against pertussis—using either a whole-cell or acellular vaccine—vaccination coverage remains below the levels recommended by the World Health Organization (WHO). According to WHO data, since 2017, vaccination coverage in the Americas has ranged from 80 to 88%, falling short of the 90% threshold set by the WHO Immunization Agenda (12). In Brazil, the DTaP vaccination coverage has declined over the years. Fortunately, since 2022, a gradual annual increase has been observed; however, coverage still remains well below the levels recommended by the WHO (Figure 5) (4).

### A shift in the epidemiological profile of *Bordetella pertussis*: not only a childhood disease

The coronavirus disease 2019 (COVID-19) pandemic may have contributed to a reduction in the prevalence of respiratory pathogens, such as *B. pertussis*, owing to public health measures, including social distancing, facial mask use, quarantine, and enhanced hand hygiene practices (13–15). These factors might have led to missed vaccination opportunities, thereby contributing to the observed decline in vaccination coverage (16, 17). Pertussis vaccination is recommended during each pregnancy and should be a part of the routine immunization schedule for healthcare professionals. It is noteworthy that misinformation regarding the vaccine is not limited to the general population, and a significant proportion of healthcare providers remain unaware of the current DTaP vaccine recommendations (18). In this context, enhancing healthcare workers’ education regarding the indications and benefits of vaccination is essential to improve immunization uptake and prevent future outbreaks.

In Brazil, *B. pertussis* infection must be reported for any individual, regardless of age or vaccination status, who presents with a dry cough lasting at least 14 days, accompanied by one or more of the following criteria: (a) paroxysmal coughing (sudden, uncontrollable episodes involving 5–10 rapid coughs in a single exhalation), (b) inspiratory whoop or post-tussive vomiting, or (c) known contact with a confirmed case of whooping cough. Laboratory confirmation is based on real-time polymerase chain reaction (RT-PCR) or culture of *B. pertussis*. Although our dataset revealed an increase in whooping cough cases in Brazil, these findings must be interpreted with caution. The broader use of molecular diagnostic tools has led to an increase in the number of confirmed cases in regions such as São Paulo and the United States (19, 20). Unfortunately, molecular testing is not universally applicable to all regions of Brazil. Many healthcare facilities rely on clinical diagnosis and bacterial culture. While the culture offers high specificity, it is limited by low sensitivity and poor negative predictive value (19, 21). RT-PCR is considered to be the diagnostic gold standard, with the *IS481* insertion sequence being considered the most commonly targeted gene, yielding approximately 93% sensitivity. However, this gene is also present in other *Bordetella* species, such as *Bordetella holmesii* and some strains of *Bordetella parapertussis* and *Bordetella bronchiseptica*, which may contribute to false-positive results (22–24). Such misdiagnoses can compromise surveillance data on the epidemiology of whooping cough and potentially affect the perceived efficacy of vaccination (24, 25).

Although pertussis is predominantly prevalent in children, our data indicate an increase in the number of cases among older individuals in Brazil. Previous studies have estimated the prevalence of pertussis to be approximately 3.5% among individuals over 18 years of age, whereas another study reported a prevalence of 22.8% among individuals over 20 years of age (26). Similarly, a Brazilian study that evaluated 192 individuals aged 10 years or older with a prolonged cough found that 5.21% (10/192) tested positive for pertussis, with eight of the positive cases occurring in individuals over 18 years of age (27). Other studies have also documented a high prevalence of *B. pertussis* infection among adults, for example, a Thai study reported a rate of 18.4% (28). It is essential to assess *B. pertussis* infection in adults because undiagnosed individuals may serve as a significant source of transmission to non-immune populations, particularly infants and children (28–33). Furthermore, studies have suggested that older individuals are more likely to experience delays in pertussis diagnosis because the disease is less commonly considered in this age group, which may hinder timely treatment and lead to complications (34).

As previously described, pertussis is a highly contagious respiratory disease caused by *B. pertussis*. Although it is more common in children, many adults can also contract the disease, particularly those who have not been vaccinated or whose immunity has waned over time. It is plausible to postulate that the increased incidence of infection among adults in our sample may be associated with the following factors (34–40):

(a) *Decreased immunity over time:* The pertussis component is included in the DTaP vaccine; however, the protection conferred by the vaccine wanes over time. Many adults may not have received a booster dose after childhood, rendering them more susceptible to infection.
(b) *Genetic adaptation: B. pertussis* can undergo minor genetic modifications over time, which may reduce the effectiveness of existing vaccines against the emerging strains.
(c) *Outbreak cycles:* Pertussis follows cyclical outbreak patterns with increased bacterial circulation in certain regions during specific periods. The transmission from adults to children plays a significant role, as infants and young children are particularly vulnerable to severe complications.
(d) *Less noticeable symptoms in adults:* In adults, pertussis symptoms are often milder and less easily recognizable. The characteristic persistent cough is frequently mistaken for other respiratory conditions, such as bronchitis or common cold.

Therefore, many adults may contract pertussis without realizing it, and, in some cases, treatment may be delayed or less effective if the disease is not properly identified. A booster dose of the DTaP vaccine is recommended for adults, particularly for those in close contact with infants or individuals with weakened immune systems.

Interestingly, in our data, White individuals were more affected by pertussis, as was the South region, which is known to have a higher proportion of White individuals compared to other regions in Brazil (41). A variety of factors may explain this finding. In Brazil, Black and *Pardos* individuals typically encounter reduced access to healthcare, a higher likelihood of experiencing mistreatment, and lower rates of private health insurance coverage, all of which may hinder the timely diagnosis and notification of pertussis cases (42–46). In contrast, a majority of the hospitalized individuals in our study were *Pardos*, which may reflect delayed diagnosis in this population, potentially leading to more severe disease, supporting the aforementioned hypothesis. A recent study also highlighted the role of race in pertussis severity; in the United States, Black/African American individuals had a 1.4-fold higher incidence of severe disease compared to White individuals [(47), pp. 2010–2017].

In addition, increasing awareness regarding the diagnosis of pertussis in adults, along with improving access to diagnostic tools, is essential to halt the current outbreak and mitigate its impact. It is well documented that pertussis outbreaks occur every 3–5 years; however, the current outbreak in Latin America may have been delayed due to the COVID-19 pandemic and is arguably among the most severe pandemic ever recorded (21, 48). Similarly, Brazil has faced several other infectious disease outbreaks in the post-COVID-19 era, such as dengue fever and mpox (formerly known as monkeypox) (49, 50), which underscores the importance of preventive measures, particularly vaccination, for endemic diseases.

### Limitations

This study is based on publicly available datasets, and the authors did not have access to the original medical records. Although the data were sourced from the Brazilian Ministry of Health, discrepancies were observed in the reported number of pertussis-related deaths across the different datasets, suggesting potential errors in data entry or reporting. Furthermore, the databases did not specify the bacterial species identified in each diagnosis, increasing the probability of misdiagnosis of other *Bordetella* species. Several patient variables included a high proportion of missing data, which may have reduced the study’s statistical power. In addition, the Brazilian Ministry of Health does not provide publicly accessible information on comorbidities, the use of invasive mechanical ventilation, or information on intensive care unit admissions. The dataset also lacks information on treatment regimens and the time elapsed from symptom onset to diagnosis. Consequently, it was not possible to determine whether the observed mortality was associated with delayed diagnosis or suboptimal antibiotic therapy. These clinical markers are essential for identifying populations at higher risk of infection and those with a greater likelihood of adverse outcomes.

### Improvement perspectives

It is imperative to improve vaccination coverage across Latin America, with a particular emphasis on neonates and young children, to mitigate the impact of pertussis. Furthermore, there is an urgent need to develop more robust surveillance systems to reduce underdiagnosis and report inaccuracies, thereby enabling the effective allocation of resources and funding to the Brazilian region’s most in need.

## Data Availability

The original contributions presented in the study are included in the article/Supplementary material, further inquiries can be directed to the corresponding author.

## Footnotes

^1^ https://www.gov.br/saude/pt-br
