# Correction: The 2024 resurgence of *Bordetella pertussis* in Brazil and a decade-long epidemiological overview

**DOI:** 10.3389/fpubh.2026.1861783

**Published:** 2026-05-06

**Authors:** Nathália Mariana Santos Sansone, Matheus Negri Boschiero, Fernando Augusto Lima Marson

**Affiliations:** 1 LunGuardian Research Group—Epidemiology of Respiratory and Infectious Diseases, São Francisco University, Bragança Paulista, Brazil; 2 Laboratory of Molecular Biology and Genetics, São Francisco University, Bragança Paulista, Brazil; 3 Laboratory of Clinical and Molecular Microbiology, São Francisco University, Bragança Paulista, Brazil; 4 Medical Resident of Infectious Diseases at the Federal University of São Paulo, São Paulo, Brazil

**Keywords:** *Bordetella pertussis*, epidemiology, incidence, pertussis, public health, vaccine

There was a mistake in Figure 1 as published. In the original version, the *y*-axis of the graph displayed incorrect year labels. In the corrected version, the years have been properly adjusted. Importantly, the values corresponding to the number of cases were accurate in the original figure; only the year labels were presented incorrectly.

**Figure 1:**
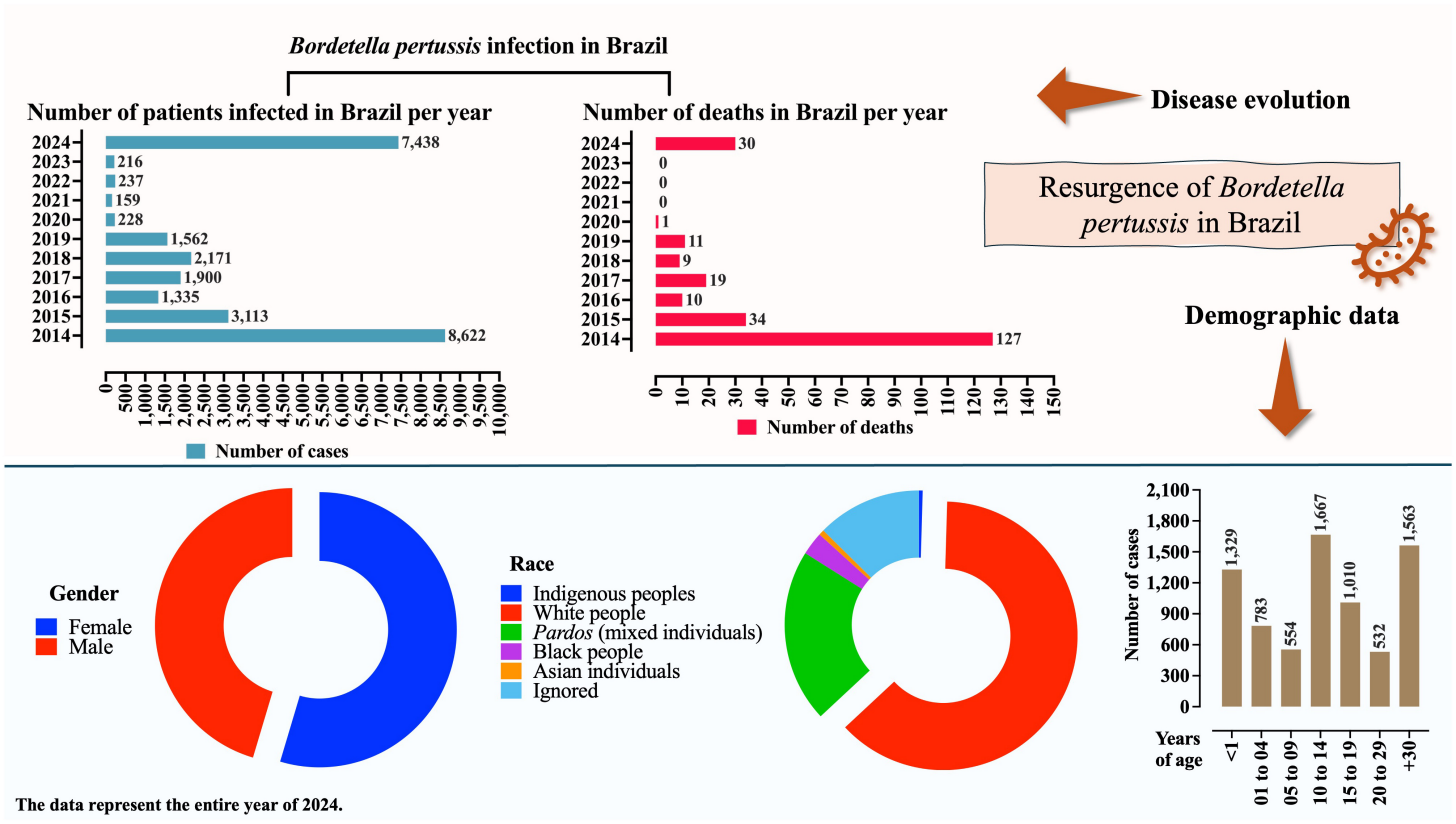
Profile of *Bordetella pertussis* infection in Brazil based on the number of cases, deaths, sex, race, and age groups. Data on sex, race, and age refer to the year 2024. Information is presented as the number of cases (*N*) or percentage. Epidemiological data on pertussis in Brazil were obtained from the Brazilian Ministry of Health (https://www.gov.br/saude/pt-br). Data were collected on 24 April 2025. Some changes may have occurred, particularly regarding the 2024 figures, due to the inclusion of newly confirmed cases.

The corrected Figure 1 appears below.

The original version of this article has been updated.

