# Supplementary material for: The 2024 resurgence of Bordetella pertussis in Brazil and a decade-long epidemiological overview
Source: Front Public Health. 2025 May 27;13:1549735. doi: 10.3389/fpubh.2025.1549735 (PMC12149204; doi:10.3389/fpubh.2025.1549735)
Supplement: Supplementary file 1 [file Table_1.docx]

**TITLE**: The 2024 Resurgence of Bordetella pertussis in Brazil and a Decade-Long Epidemiological Overview

**RUNNING TITLE**: Whooping cough

| **Table 1.** Description of the epidemiological profile of pertussis (*Bordetella pertussis* infection) cases reported in Brazil over the past ten years (period from 2014 to 2024). | | | | | | | | | | | | |
| --- | --- | --- | --- | --- | --- | --- | --- | --- | --- | --- | --- | --- |
| **Year** | **2014** | **2015** | **2016** | **2017** | **2018** | **2019** | **2020** | **2021** | **2022** | **2023** | **2024** | **Total** |
| **Number of cases** | 8,622 | 3,113 | 1,335 | 1,900 | 2,171 | 1,562 | 228 | 159 | 237 | 216 | 7,438 | 26,981 |
| **Number of deaths** | 127 | 34 | 10 | 19 | 9 | 11 | 1 | 0 | 0 | 0 | 30 | 241 |
| **Incidence per 100,000 inhabitants** | 4.29 | 1.54 | 0.66 | 0.93 | 1.05 | 0.75 | 0.11 | 0.08 | 0.11 | 0.10 | 3.50 | - |
| **Lethality (%)** | 1.47 | 1.09 | 0.75 | 1.00 | 0.41 | 0.70 | 0.44 | - | - | - | 0.40 | - |
| **Sex** |  |  |  |  |  |  |  |  |  |  |  |  |
| Female | 4,820 (55.9) | 1,724 (55.4) | 754 (56.5) | 1,047 (55.1) | 1,211 (55.8) | 844 (54.0) | 122 (53.5) | 85 (53.5) | 115 (48.5) | 123 (56.9) | 4,063 (54.6) | 14,897 (55.2) |
| Male | 3,798 (55.1) | 1,389 (44.6) | 581 (43.5) | 853 (44.9) | 959 (44.2) | 717 (45.9) | 106 (46.5) | 74 (46.5) | 122 (51.5) | 93 (43.1) | 3,375 (45.4) | 12,078 (44.7) |
| **Race** |  |  |  |  |  |  |  |  |  |  |  |  |
| White people | 4,030 (46.6) | 1,371 (44.0) | 628 (47.0) | 1,008 (53.1) | 1,028 (47.4) | 587 (37.6) | 84 (36.4) | 49 (30.8) | 81 (34.2) | 99 (45.8) | 4,657 (62.6) | 13,621 (50.5) |
| Black people | 255 (3.0) | 89 (2.9) | 44 (3.3) | 51 (2.7) | 52 (2.4) | 53 (3.4) | 6 (2.6) | 6 (3.8) | 10 (4.2) | 9 (4.2) | 207 (2.8) | 782 (2.9) |
| *Pardos* (Mixed individuals) | 2,798 (32.5) | 1,143 (36.7) | 440 (33.0) | 545 (28.6) | 755 (34.7) | 714 (45.7) | 98 (43.0) | 77 (48.4) | 109 (46.0) | 76 (35.2) | 1,553 (20.9) | 8,308 (30.8) |
| Asian individuals | 47 (0.6) | 10 (0.3) | 1 (<0.1) | 7 (0.4) | 15 (0.7) | 5 (0.3) | 2 (0.9) | - | - | 2 (0.9) | 49 (0.7) | 138 (0.5) |
| Indigenous peoples | 79 (0.9) | 36 (1.20 | 8 (0.6) | 8 (0.4) | 9 (0.4) | 25 (1.6) | 4 (1.8) | 1 (0.6) | 1 (0.4) | 1 (0.5) | 34 (0.5) | 206 (0.8) |
| Ignored | 1,413 (16.4) | 464 (14.9) | 214 (16.0) | 281 (14.8) | 312 (14.4) | 178 (11.4) | 35 (15.4) | 26 (16.4) | 36 (15.2) | 29 (13.4) | 938 (12.6) | 3,926 (14.5) |
| **Age (years of age)** |  |  |  |  |  |  |  |  |  |  |  |  |
| <1 | 5,129 (59.5) | 1,937 (62.2) | 850 (63.7) | 1,068 (56.2) | 1,134 (52.2) | 819 (52.4) | 127 (55.7) | 100 (62.9) | 133 (56.1) | 125 (57.8) | 1,329 (17.8) | 12,751 (47.3) |
| 01 to 04 | 1,229 (14.2) | 435 (14.5) | 195 (14.6) | 303 (15.9) | 381 (17.5) | 324 (20.7) | 44 (19.2) | 35 (22.0) | 79 (33.3) | 36 (16.6) | 783 (10.5) | 3,844 (14.3) |
| 05 to 09 | 849 (9.8) | 280 (9.0) | 106 (7.9) | 198 (10.4) | 185 (8.5) | 105 (6.7) | 10 (4.4) | 6 (3.7) | 15 (6.3) | 17 (7.9) | 554 (7.4) | 2,325 (8.6) |
| 10 to 14 | 425 (4.9) | 138 (4.4) | 42 (3.1) | 126 (6.6) | 176 (8.1) | 128 (8.2) | 9 (3.9) | 2 (1.3) | 1 (0.4) | 18 (8.3) | 1,667 (22.4) | 2,732 (10.1) |
| 15 to 19 | 133 (1.5) | 48 (1.5) | 20 (1.5) | 35 (1.8) | 40 (1.8) | 26 (1.7) | 4 (1.7) | 2 (1.3) | 2 (0.8) | 3 (1.4) | 1,010 (13.5) | 1,323 (4.9) |
| 20 to 29 | 298 (3.4) | 91 (2.9) | 40 (3.0) | 40 (2.1) | 48 (2.2) | 41 (2.6) | 6 (2.6) | 4 (2.4) | 2 (0.8) | 2 (0.9) | 532 (7.1) | 1,104 (4.1) |
| +30 | 555 (6.4) | 184 (5.9) | 82 (6.1) | 130 (6.8) | 206 (9.5) | 118 (7.6) | 28 (12.2) | 10 (6.3) | 5 (2.1) | 15 (6.9) | 1,563 (21.0) | 2,896 (10.7) |
| **Place of residence** |  |  |  |  |  |  |  |  |  |  |  |  |
| Urban | 7,467 (86.6) | 2,694 (86.5) | 1,174 (87.9) | 1,686 (88.7) | 1,917 (88.3) | 1,374 (88.0) | 198 (86.8) | 142 (89.3) | 213 (88.9) | 196 (90.7) | 6,805 (91.5) | 23,866 (88.4) |
| Rural | 802 (9.3) | 294 (9.4) | 88 (6.6) | 139 (7.3) | 158 (7.3) | 137 (8.8) | 17 (7.5) | 10 (6.3) | 18 (7.6) | 8 (3.7) | 276 (3.7) | 1,947 (7.2) |
| Peri-urban | 67 (0.8) | 22 (0.7) | 5 (0.4) | 12 (0.6) | 18 (0.8) | 10 (0.6) | - | 1 (0.6) | - | - | 57 (0.8) | 192 (0.7) |
| Ignored | 286 (3.3) | 103 (3.3) | 68 (5.1) | 63 (3.3) | 78 (3.6) | 41 (2.6) | 13 (5.7) | 6 (3.7) | 6 (2.5) | 12 (5.6) | 300 (4.0) | 976 (3.6) |

The data are presented as the number of cases (N) and percentages (%). Incidence is presented as the number of cases per 100,000 inhabitants. Epidemiological data on pertussis in Brazil was obtained from the Brazilian Ministry of Health (<https://www.gov.br/saude/pt-br>). Data were collected on April 24, 2025 — some changes may still occur, particularly regarding 2024 figures, due to the inclusion of newly confirmed cases.

| **Table 2.** Description of the epidemiological profile of pertussis cases requiring hospitalization in Brazil over the past ten years (period from 2014 to 2024). | | | | | | | | | | | | |
| --- | --- | --- | --- | --- | --- | --- | --- | --- | --- | --- | --- | --- |
| **Year** | **2014** | **2015** | **2016** | **2017** | **2018** | **2019** | **2020** | **2021** | **2022** | **2023** | **2024** | **Total** |
| **Number of hospitalizations** | 4,926 | 2,492 | 1,188 | 1,221 | 1,460 | 1,200 | 318 | 192 | 285 | 236 | 739 | 14,257 |
| **Number of hospitalizations per 100,000 inhabitants** | 2.46 | 1.23 | 0.58 | 0.59 | 0.70 | 0.57 | 0.15 | 0.09 | 0.13 | 0.11 | 0.34 | - |
| **Sex** |  |  |  |  |  |  |  |  |  |  |  |  |
| Male | 2,307 (46.8) | 1,164 (46.7) | 577 (48.6) | 600 (49.1) | 675 (46.2) | 596 (49.7) | 148 (46.5) | 90 (46.9) | 138 (48.4) | 105 (44.5) | 363 (49.1) | 6,763 (47.4) |
| Female | 2,619 (53.2) | 1,328 (53.3) | 611 (51.4) | 621 (50.9) | 785 (53.8) | 604 (50.3) | 170 (53.5) | 102 (53.1) | 147 (51.6) | 131 (55.5) | 376 (50.9) | 7,494 (52.6) |
| **Age (years of age)** |  |  |  |  |  |  |  |  |  |  |  |  |
| <1 | 4,333 (88.0) | 2,184 (87.6) | 1,027 (86.4) | 1,034 (84.7) | 1,198 (82.1) | 949 (79.1) | 250 (78.6) | 165 (85.9) | 226 (79.3) | 193 (81.8) | 549 (74.2) | 12,108 (84.9) |
| 1 to 4 | 364 (7.4) | 197 (7.9) | 115 (9.7) | 119 (9.7) | 181 (12.4) | 199 (16.6) | 49 (15.4) | 14 (7.3) | 38 (13.3) | 17 (7.2) | 112 (15.2) | 1,405 (9.8) |
| 5 to 9 | 124 (2.5) | 55 (2.2) | 17 (1.4) | 32 (2.6) | 23 (1.6) | 20 (1.7) | 4 (1.3) | 6 (3.1) | 6 (2.1) | 5 (2.1) | 16 (2.2) | 304 (2.1) |
| 10 to 14 | 41 (0.8) | 20 (0.8) | 6 (0.5) | 15 (1.2) | 24 (1.6) | 12 (1.0) | 4 (1.3) | - | - | 2 (0.8) | 14 (1.9) | 138 (1.0) |
| 15 to 19 | 9 (0.2) | 7 (0.3) | 1 (0.1) | 3 (0.2) | 4 (0.3) | 5 (0.4) | 2 (0.6) | 1 (0.5) | - | - | 8 (1.1) | 40 (0.3) |
| 20 to 29 | 11 (0.2) | 6 (0.2) | 1 (0.1) | 4 (0.3) | 4 (0.3) | 2 (0.2) | 2 (0.6) | - | 4 (1.4) | 1 (0.4) | 4 (0.5) | 39 (0.3) |
| >30 years | 44 (0.9) | 23 (0.9) | 21 (1.8) | 14 (1.1) | 26 (1.8) | 13 (1.1) | 7 (2.2) | 6 (3.1) | 11 (3.9) | 18 (7.6) | 36 (4.9) | 219 (1.5) |
| **Race** |  |  |  |  |  |  |  |  |  |  |  |  |
| White people | 1,398 (28.4) | 688 (27.6) | 394 (33.2) | 434 (35.5) | 457 (31.3) | 254 (21.2) | 69 (21.7) | 39 (20.3) | 75 (26.3) | 71 (30.1) | 312 (42.2) | 4,191 (29.3) |
| Black people | 53 (1.1) | 36 (1.4) | 17 (1.4) | 23 (1.9) | 16 (1.1) | 14 (1.2) | 5 (1.6) | 1 (0.5) | 6 (2.1) | 6 (2.5) | 25 (3.3) | 202 (1.4) |
| *Pardos* (Mixed individuals) | 1,624 (33.0) | 850 (34.1) | 375 (31.6) | 370 (30.3) | 524 (35.9) | 541 (45.1) | 152 (47.8) | 67 (34.9) | 120 (42.1) | 150 (63.6) | 394 (53.3) | 5,167 (36.2) |
| Asian individuals | 13 (0.3) | 6 (0.2) | 2 (0.2) | 4 (0.3) | 7 (0.5) | 7 (0.6) | 1 (0.3) | 1 (0.5) | 1 (0.4) | 2 (0.8) | 5 (0.7) | 49 (0.3) |
| Indigenous peoples | 24 (0.5) | 12 (0.5) | 5 (0.4) | 4 (0.3) | 3 (0.2) | 12 (1.0) | 1 (0.3) | - | 2 (0.7) | 1 (0.4) | 3 (0.7) | 61 (0.5) |
| Ignored | 1,814 (36.8) | 900 (36.1) | 395 (33.2) | 386 (31.6) | 453 (31.0) | 372 (31.0) | 90 (28.3) | 84 (43.8) | 81 (28.4) | 6 (2.5) | - | 4,581 (33.1) |

The data are presented as the number of cases (N) and the percentages (%). Incidence is presented as the number of cases per 100,000 inhabitants. Epidemiological data on pertussis in Brazil was obtained from the Brazilian Ministry of Health (<https://www.gov.br/saude/pt-br>). Data were collected on April 24, 2025 — some changes may still occur, particularly regarding 2024 figures, due to the inclusion of newly confirmed cases.

| **Table 3.** Number of whooping cough cases by macro-regions and federal units of Brazil. | | | | | | | | | | | | |
| --- | --- | --- | --- | --- | --- | --- | --- | --- | --- | --- | --- | --- |
| **Regions and Federative Unit** | **2014** | **2015** | **2016** | **2017** | **2018** | **2019** | **2020** | **2021** | **2022** | **2023** | **2024** | **Total** |
| **North** | **481** | **235** | **100** | **98** | **81** | **71** | **16** | **10** | **4** | **4** | **40** | **1,268** |
| Acre | 80 | 16 | 0 | 5 | 3 | 3 | 0 | 0 | 0 | 0 | 0 | 107 |
| Amapá | 48 | 5 | 5 | 7 | 2 | 0 | 0 | 1 | 0 | 0 | 0 | 68 |
| Amazonas | 68 | 115 | 63 | 42 | 17 | 14 | 4 | 4 | 1 | 1 | 26 | 355 |
| Pará | 104 | 26 | 4 | 5 | 32 | 18 | 5 | 3 | 0 | 1 | 5 | 331 |
| Rondônia | 78 | 27 | 7 | 6 | 5 | 4 | 1 | 0 | 1 | 2 | 3 | 134 |
| Roraima | 9 | 4 | 5 | 21 | 5 | 21 | 0 | 0 | 0 | 0 | 4 | 69 |
| Tocantins | 94 | 42 | 16 | 12 | 17 | 11 | 6 | 2 | 2 | 0 | 2 | 204 |
| **Northeast** | **2,938** | **967** | **339** | **395** | **690** | **761** | **78** | **71** | **128** | **83** | **258** | **6,708** |
| Alagoas | 221 | 34 | 19 | 14 | 36 | 23 | 1 | 3 | 5 | 2 | 1 | 359 |
| Bahia | 621 | 132 | 34 | 37 | 153 | 80 | 10 | 11 | 18 | 4 | 95 | 1,195 |
| Ceará | 184 | 68 | 15 | 20 | 15 | 39 | 16 | 0 | 2 | 2 | 23 | 384 |
| Maranhão | 160 | 83 | 12 | 14 | 26 | 23 | 2 | 1 | 1 | 4 | 5 | 331 |
| Paraíba | 56 | 17 | 0 | 9 | 1 | 4 | 0 | 1 | 3 | 0 | 2 | 93 |
| Pernambuco | 1,217 | 410 | 198 | 253 | 407 | 528 | 43 | 53 | 85 | 65 | 118 | 3,377 |
| Piauí | 364 | 151 | 31 | 30 | 27 | 32 | 2 | 0 | 1 | 0 | 0 | 638 |
| Rio Grande do Norte | 95 | 68 | 30 | 15 | 22 | 31 | 4 | 2 | 10 | 3 | 12 | 292 |
| Sergipe | 20 | 4 | 0 | 3 | 3 | 1 | 0 | 0 | 3 | 3 | 2 | 39 |
| **Southeast** | **2,920** | **1071** | **558** | **736** | **808** | **432** | **79** | **41** | **37** | **75** | **3,134** | **9,891** |
| Espiríto Santo | 285 | 167 | 106 | 92 | 73 | 27 | - | - | - | - | - | 750 |
| Minas Gerais | 360 | 256 | 112 | 181 | 233 | 182 | 26 | 15 | 14 | 14 | 847 | 2,240 |
| Rio de Janeiro | 111 | 91 | 79 | 50 | 40 | 50 | 15 | 7 | 5 | 8 | 599 | 1,055 |
| São Paulo | 2,164 | 557 | 261 | 413 | 462 | 173 | 38 | 19 | 18 | 53 | 1,688 | 5,846 |
| **South** | **1,477** | **539** | **274** | **590** | **417** | **197** | **42** | **26** | **50** | **42** | **3,579** | **7,233** |
| Paraná | 973 | 294 | 118 | 150 | 178 | 101 | 26 | 9 | 5 | 17 | 2,776 | 4,647 |
| Rio Grande do Sul | 260 | 128 | 110 | 320 | 168 | 65 | 8 | 13 | 39 | 23 | 451 | 1,585 |
| Santa Catarina | 244 | 117 | 46 | 120 | 71 | 31 | 8 | 4 | 6 | 2 | 352 | 1,001 |
| **Central-West** | **804** | **296** | **62** | **81** | **175** | **101** | **13** | **11** | **18** | **12** | **426** | **1,999** |
| Federal District | 243 | 99 | 31 | 35 | 75 | 54 | 5 | 8 | 7 | 5 | 259 | 821 |
| Goiás | 220 | 71 | 8 | 22 | 32 | 31 | 1 | 3 | 4 | 0 | 96 | 488 |
| Mato Grosso | 160 | 72 | 17 | 6 | 31 | 11 | 3 | 0 | 7 | 2 | 38 | 347 |
| Mato Grosso do Sul | 181 | 54 | 6 | 18 | 37 | 5 | 4 | 0 | 0 | 5 | 33 | 343 |
| **Total** | 8,620 | 3,108 | 1,333 | 1,900 | 2,171 | 1,562 | 228 | 159 | 237 | 216 | 7,437 | 26,971 |

The data are presented as the number of cases (N) and the percentages (%). Epidemiological data on pertussis in Brazil was obtained from the Brazilian Ministry of Health (https://www.gov.br/saude/pt-br). Data were collected on April 24, 2025 — some changes may still occur, particularly regarding 2024 figures, due to the inclusion of newly confirmed cases. -, the data were not available for collection.

| **Table 4.** Incidence of whooping cough cases per 100,000 inhabitants by macro-regions and federal units of Brazil. | | | | | | | | | | | |
| --- | --- | --- | --- | --- | --- | --- | --- | --- | --- | --- | --- |
| **Incidence per 100,000 inhabitants** | **2014** | **2015** | **2016** | **2017** | **2018** | **2019** | **2020** | **2021** | **2022** | **2023** | **2024** |
| **North** | **2.83** | **1.36** | **0.57** | **0.56** | **0.46** | **0.40** | **0.09** | **0.06** | **0.02** | **0.02** | **0.21** |
| Acre | 9.81 | 1.94 | 0.00 | 0.59 | 0.35 | 0.35 | 0.00 | 0.00 | 0.00 | 0.00 | 0.00 |
| Amapá | 6.52 | 0.67 | 0.66 | 0.91 | 0.26 | 0.00 | 0.00 | 0.13 | 0.00 | 0.00 | 0.00 |
| Amazonas | 1.79 | 2.98 | 1.61 | 1.06 | 0.42 | 0.34 | 0.10 | 0.10 | 0.02 | 0.02 | 0.61 |
| Pará | 1.29 | 0.32 | 0.05 | 0.06 | 0.38 | 0.21 | 0.06 | 0.04 | 0.00 | 0.01 | 0.06 |
| Rondônia | 4.72 | 1.62 | 0.42 | 0.36 | 0.29 | 0.23 | 0.06 | 0.00 | 0.06 | 0.12 | 0.17 |
| Roraima | 1.73 | 0.75 | 0.91 | 3.72 | 0.85 | 3.40 | 0.00 | 0.00 | 0.00 | 0.00 | 0.56 |
| Tocantins | 6.46 | 2.85 | 1.08 | 0.80 | 1.13 | 0.72 | 0.39 | 0.13 | 0.13 | 0.00 | 0.13 |
| **Northeast** | **5.35** | **1.75** | **0.61** | **0.71** | **1.23** | **1.35** | **0.14** | **0.13** | **0.23** | **0.15** | **0.45** |
| Alagoas | 6.97 | 1.07 | 0.60 | 0.44 | 1.13 | 0.72 | 0.03 | 0.09 | 0.16 | 0.06 | 0.03 |
| Bahia | 4.31 | 0.91 | 0.23 | 0.25 | 1.05 | 0.54 | 0.07 | 0.07 | 0.12 | 0.03 | 0.64 |
| Ceará | 2.10 | 0.77 | 0.17 | 0.22 | 0.17 | 0.43 | 0.18 | 0.00 | 0.02 | 0.02 | 0.25 |
| Maranhão | 2.36 | 1.22 | 0.18 | 0.20 | 0.38 | 0.33 | 0.03 | 0.01 | 0.01 | 0.06 | 0.07 |
| Paraíba | 1.43 | 0.43 | 0.00 | 0.23 | 0.03 | 0.10 | 0.00 | 0.02 | 0.07 | 0.00 | 0.05 |
| Pernambuco | 13.29 | 4.45 | 2.14 | 2.72 | 4.35 | 5.62 | 0.46 | 0.56 | 0.90 | 0.68 | 1.24 |
| Piauí | 11.24 | 4.64 | 0.95 | 0.91 | 0.82 | 0.96 | 0.06 | 0.00 | 0.03 | 0.00 | 0.00 |
| Rio Grande do Norte | 2.88 | 2.05 | 0.90 | 0.45 | 0.65 | 0.91 | 0.12 | 0.06 | 0.29 | 0.09 | 0.35 |
| Sergipe | 0.92 | 0.18 | 0.00 | 0.14 | 0.14 | 0.05 | 0.00 | 0.00 | 0.13 | 0.13 | 0.09 |
| **Southeast** | **3.44** | **1.25** | **0.65** | **0.85** | **0.93** | **0.49** | **0.09** | **0.05** | **0.04** | **0.09** | **3.54** |
| Espiríto Santo | 7.51 | 4.36 | 2.74 | 2.36 | 1.85 | 0.68 | - | - | - | - | - |
| Minas Gerais | 1.77 | 1.25 | 0.54 | 0.88 | 1.12 | 0.87 | 0.12 | 0.07 | 0.07 | 0.07 | 3.97 |
| Rio de Janeiro | 0.66 | 0.54 | 0.46 | 0.29 | 0.23 | 0.29 | 0.09 | 0.04 | 0.03 | 0.05 | 3.48 |
| São Paulo | 4.93 | 1.26 | 0.59 | 0.92 | 1.03 | 0.38 | 0.08 | 0.04 | 0.04 | 0.04 | 0.12 |
| **South** | **5.13** | **1.86** | **0.94** | **2.00** | **1.40** | **0.66** | **0.14** | **0.09** | **0.16** | **0.14** | **11.50** |
| Paraná | 8.84 | 2.65 | 1.05 | 1.33 | 1.57 | 0.88 | 0.23 | 0.08 | 0.04 | 0.15 | 23.48 |
| Rio Grande do Sul | 2.36 | 1.16 | 0.99 | 2.87 | 1.50 | 0.58 | 0.07 | 0.12 | 0.35 | 0.21 | 4.02 |
| Santa Catarina | 3.60 | 1.70 | 0.66 | 1.68 | 0.98 | 0.42 | 0.11 | 0.05 | 0.08 | 0.03 | 4.37 |
| **Midwest** | **5.29** | **1.92** | **0.40** | **0.51** | **1.09** | **0.62** | **0.08** | **0.07** | **0.11** | **0.07** | **2.50** |
| Federal District | 8.79 | 3.54 | 1.10 | 1.23 | 2.61 | 1.86 | 0.17 | 0.27 | 0.24 | 0.17 | 8.68 |
| Goiás | 3.37 | 1.07 | 0.12 | 0.32 | 0.47 | 0.44 | 0.01 | 0.04 | 0.06 | 0.00 | 1.31 |
| Mato Grosso | 4.89 | 2.16 | 0.50 | 0.18 | 0.89 | 0.31 | 0.08 | 0.00 | 0.19 | 0.05 | 0.99 |
| Mato Grosso do Sul | 6.91 | 2.04 | 0.22 | 0.66 | 1.35 | 0.18 | 0.14 | 0.00 | 0.00 | 0.17 | 1.14 |
| **Total** | **4.29** | **1.54** | **0.65** | **0.93** | **1.05** | **0.75** | **0.11** | **0.08** | **0.11** | **0.10** | **3.50** |

The data are presented as the number of cases (N) and the percentages (%). Incidence is presented as the number of cases per 100,000 inhabitants. Epidemiological data on pertussis in Brazil was obtained from the Brazilian Ministry of Health (https://www.gov.br/saude/pt-br). Data were collected on April 24, 2025 — some changes may still occur, particularly regarding 2024 figures, due to the inclusion of newly confirmed cases. -, the data were not available for collection.
